# Overcoming drug resistance in castrate-resistant prostate cancer: current mechanisms and emerging therapeutic approaches

**DOI:** 10.20517/cdr.2024.173

**Published:** 2025-02-19

**Authors:** Adam Khorasanchi, Feng Hong, Yuanquan Yang, Eric A. Singer, Peng Wang, Mingjia Li, Linghua Zheng, Paul Monk, Amir Mortazavi, Lingbin Meng

**Affiliations:** ^1^Division of Hospital Medicine, The Ohio State University Comprehensive Cancer Center, Columbus, OH 43210, USA.; ^2^Pelotonia Institute for Immuno-Oncology, The Ohio State University, Columbus, OH 43210, USA.; ^3^Division of Medical Oncology, Department of Internal Medicine, The Ohio State University Comprehensive Cancer Center, Columbus, OH 43210, USA.; ^4^Division of Urologic Oncology, The Ohio State University Comprehensive Cancer Center, Columbus, OH 43210, USA.

**Keywords:** Drug resistance, androgen receptor, castration-resistant prostate cancer, androgen deprivation therapy, chemotherapy, immunotherapy, targeted therapies, radionuclide therapy

## Abstract

Metastatic castration-resistant prostate cancer (mCRPC) is driven by a complex network of resistance mechanisms against standard-of-care therapies, resulting in poor long-term outcomes. This review offers a uniquely comprehensive and integrative perspective on these resistance pathways, systematically examining both androgen receptor (AR)-dependent factors (including AR overexpression, point mutations, glucocorticoid receptor signaling, splice variants, post-translational modifications, altered coregulators, and intratumoral hormone biosynthesis) and AR-independent pathways (such as neuroendocrine differentiation, lineage plasticity, and alternative growth factor signaling). We also highlight resistance mechanisms influencing immunotherapy, chemotherapy, radiopharmaceutical therapy and targeted therapy. By synthesizing emerging insights across these domains, this review not only clarifies the underlying biology of mCRPC resistance but also identifies key leverage points for more effective interventions. Building on this foundation, we propose a forward-looking framework for overcoming mCRPC drug resistance, emphasizing the importance of biomarker-guided patient selection, combination strategies that simultaneously target multiple resistance mechanisms, and novel therapies under investigation. These recommendations are intended to guide future clinical trial designs and research priorities that move beyond incremental improvements. Ultimately, this comprehensive synthesis aims to serve as a resource for clinicians and researchers to accelerate the development of durable, precision-based treatment strategies in mCRPC.

## INTRODUCTION

Prostate cancer (PC) is the second most common cancer diagnosed in men and remains a significant source of morbidity and mortality worldwide^[1]^. Approximately 8% of patients present with metastatic disease but account for 50% of PC-related mortality, with a 5-year survival rate of only 36.6%^[2,3]^. Over the past decade, incidence rates of metastatic PC have also risen dramatically, particularly among men over 75 years of age and non-Hispanic whites^[4]^.

The current standard first-line treatment for metastatic castrate-sensitive PC (mCSPC) is androgen deprivation therapy (ADT)^[5]^. Treatment intensification with either androgen receptor signaling inhibitors (ARSIs)^[6]^ or docetaxel^[7,8]^ in combination with ADT has demonstrated improved survival outcomes. However, disease progression to castration-resistant prostate cancer (CRPC) occurs in 10%-20% of patients within 5 years of initial therapy, with 84% of these cases presenting with metastatic disease^[9]^. CRPC is characterized by disease progression despite castrate testosterone levels. The Prostate Cancer Working Group 3 (PCWG3) defines progression based on three criteria: biochemical [prostate-specific antigen (PSA) increase ≥ 25% and ≥ 2 ng/mL above nadir], radiographic (per RECIST criteria), or clinical (worsening pain or quality of life)^[10]^.

Recent advances in understanding PC tumor biology have led to several new therapeutic options, including radioactive calcium mimetics targeting bone metastases [Radium-223 (Ra-223)], immunotherapy (sipuleucel-T), poly ADP-ribose polymerase inhibitors (PARPis), and lutetium (177Lu) vipivotide tetraxetan^[11]^. Despite these significant advances in treatment, metastatic castration-resistant prostate cancer (mCRPC) remains incurable and lethal. One important prognostic factor in mCRPC is the site of metastatic disease involvement^[12]^. For instance, patients with bony metastases have a median OS of 21 months compared with 16 months in those with visceral disease^[13,14]^. Additionally, among patients who have osseous metastatic disease, bone-related parameters such as serum alkaline phosphatase levels and the occurrence of skeletal-related events have been shown to significantly influence survival outcomes^[15]^. Several genetic alterations have been identified in mCRPC, including the androgen receptor (*AR*) gene (the primary driver of disease progression), *RB1* (loss of tumor suppressor associated with worse prognosis), and DNA damage response (*BRCA 1/2*, *ATM*) genes, which serve as the basis for current drug targets^[5]^.

This review provides a comprehensive overview of the diverse mechanisms contributing to drug resistance in mCRPC. Furthermore, it explores promising therapeutic strategies currently being investigated in clinical trials that may overcome drug resistance and enhance patient outcomes.

## AR-DEPENDENT RESISTANCE MECHANISMS IN PC

### AR structure and overexpression/amplification

The AR signaling pathway plays a crucial role in PC cell proliferation and survival^[5,16]^. The AR protein is composed of three major domains: an N-terminal domain (NTD), which interacts with various coregulators, a ligand binding domain (LBD), which is the site of androgen binding, and a DNA binding domain (DBD), which binds to AR response elements following activation to regulate the transcription of associated genes^[17]^. When testosterone or dihydrotestosterone (DHT) binds to the AR, it undergoes a conformational change that enables dimerization, nuclear localization, and transcription of genes that promote PC growth and survival^[18]^. This pathway underlies the use of ADT as a first-line treatment for castrate-sensitive PC (CSPC)^[19]^. The purpose of ADT is to lower serum androgen to castrate levels to prevent AR activation^[5]^. Unfortunately, despite ADT, patients often still progress to CRPC^[20]^. One reason for this is AR overexpression, which increases AR signaling activity by sensitizing PC cells to very low levels of circulating androgen. Potential contributors include AR gene amplification, epigenetic modifications at AR enhancer sites, dysregulation of AR coactivators/corepressors, and enhanced AR protein stability^[21]^.

### AR point mutations

AR point mutations are present in 10%-20% of patients with CRPC^[22]^. These gain-of-function missense mutations alter the AR ligand-binding pocket and typically arise following treatment with ARSI. ARSI agents block the AR LBD, thereby displacing androgens and preventing its activation. First-generation ARSI antagonists include bicalutamide, flutamide, and nilutamide, while second-generation ARSIs exhibit a higher affinity for the AR LBD, including enzalutamide, apalutamide, and darolutamide. Most AR point mutations occur in the LBD (e.g., T878A, L702H, W742C, and H875Y)^[23]^. These mutations can result in enhanced sensitivity to low androgen levels and broader AR ligand specificity. Additionally, certain mutations (e.g., T878A) can convert AR antagonists like enzalutamide into agonists, leading to ARSI resistance^[17,24]^.

### Glucocorticoid receptor signaling

Under physiologic conditions, the glucocorticoid receptor (GR) and AR share common transcriptional target genes, and GR expression is suppressed through the AR signaling pathway. However, in PC treated with ADT (AR blockade), GR expression can rise and drive transcription of AR target genes contributing to PC progression^[25]^.

### AR splice variants

AR splice variants (AR-Vs) encode a truncated AR protein missing the C-terminal LBD. Lacking this domain creates a constitutively active transcription factor able to drive downstream AR signaling pathways in a ligand-independent manner, further contributing to ARSI resistance^[26]^. Among these, AR-V1 and AR-V7 are the most common variants in CRPC. Notably, AR-V7 positivity is a poor prognostic indicator, associated with shorter progression-free (PFS) and overall survival (OS) in CRPC^[27,28]^. Previous studies suggest patients with AR-V7-positive mCRPC may benefit from taxane chemotherapy rather than ARSI^[29]^. Its role as a drug-resistance biomarker continues to be investigated, and two clinical trials (NCT03123978, NCT02807805) are evaluating AR-V7 as a potential drug target^[30,31]^.

### AR post-translational modifications

Post-translational modifications of AR, including serine/threonine and tyrosine phosphorylation, acetylation, methylation, and ubiquitination, represent routes to drug resistance. Collectively, these changes promote increased AR activity through enhanced protein stabilization, nuclear localization, and transcriptional regulation^[32]^. AR protein phosphorylation accounts for the majority of post-translational modifications in PC^[33]^. For instance, serine 81 (S81) phosphorylation in the AR NTD leads to increased transcriptional activity and is mediated by cyclin-dependent kinases (CDKs)^[34]^. Moreover, S81 and S213 phosphorylation correlates with worse survival outcomes following PC recurrence^[35]^. AR acetylation, facilitated by acetyltransferases, such as coactivators p300/PCAF, also serves to promote PC growth, and overexpression of these enzymes has been linked to tumor progression^[36]^. Next, protein methylation via protein methyltransferases (PNMT) can also contribute to PC growth; for example, PRMT5 normally catalyzes the methylation of arginine 761, and its dysregulation has been shown to drive CRPC development^[37]^. Finally, ubiquitination is another key process in PC development and progression. Through a cascade, the ubiquitin (Ub)-activating enzymes (E1-E3) bind and transfer Ub to a substrate, resulting in post-translational modification. In PC, ubiquitination facilitates growth and progression to mCRPC by promoting the epithelial-mesenchymal transition (EMT), mediating AR stability, maintaining cancer stem cell populations, regulating energy metabolism, and affecting cell cycle progression^[38]^.

### AR coregulators

Numerous coregulators modulate AR transcriptional activity through epigenetic regulation, acting as molecular drivers, influencing RNA splicing, or stimulating transcriptional pathways. When their activity becomes dysregulated, it can promote further disease progression in CRPC. Major growth-promoting coactivators include CBP/P300, p160/SRC, and GATA2, while NcoR1 and NcoR2 function as growth-inhibiting corepressors^[24]^. For instance, reduced NcoR2 expression correlates with worse survival after ADT, and has been linked to gene expression patterns consistent with neuroendocrine PC (NEPC) and DNA hypermethylation^[39]^.

### Intratumoral androgen and steroid hormone biosynthesis

Another mechanism by which CRPC develops drug resistance is increased intratumoral androgen and steroid hormone biosynthesis. Tumor cells circumvent the lack of circulating androgens by elevating 5 alpha (α)-reductase, which converts dehydroepiandrosterone and androstenedione to DHT, leading to enhanced AR activation^[24]^. An overall rise in intratumoral androgen synthesis, as well as greater conversion of dehydroepiandrosterone-sulfate (DHEA-S) to DHEA in the adrenal gland, can reduce ARSI effectiveness and further contribute to drug resistance^[21,40]^.

## IMMUNOTHERAPY RESISTANCE MECHANISMS IN PC

### Challenges of immunotherapy in mCRPC

Immune checkpoint inhibitors (ICIs) have led to significant improvements in OS in solid malignancies, including melanoma, bladder, and lung cancer^[41]^. Since the approval of sipuleucel-T in 2010, the first immunotherapy shown to improve OS (though not PFS) in mCRPC, there has been growing interest in developing effective immunotherapeutic agents for mCRPC^[42,43]^. However, the efficacy of immunotherapy in mCRPC has been notably limited^[44]^. The reasons are likely multifactorial. First, mCRPC is characterized by a low tumor mutational burden (TMB), resulting in fewer neoantigens that stimulate an effective antitumor immune response^[45]^. Second, mCRPC has traditionally been described as a “cold tumor” with scarce T cell infiltration, due to an immunosuppressive tumor microenvironment (TME) comprised of tumor-associated macrophages (TAMs), myeloid-derived suppressor cells (MDSCs), T-regulatory cells (Tregs), and cytokines such as transforming growth factor beta (TGF-β) and interleukin-10 (IL-10)^[46-48]^. Several studies have further characterized the immunosuppressive microenvironment of PC using single-cell RNA sequencing and spatial transcriptomics^[49-51]^. For instance, one study evaluated 19 PC samples from treatment-naïve cases post-prostatectomy, comparing them to controls with matched samples from 15/19 patients with normal tissue adjacent to their tumor, and from 4 patients without PC. The study found that the immune microenvironment in PC was characterized by suppressive myeloid cells, exhausted T cells, and elevated endothelial angiogenic activity within the stroma^[49]^. Long-term ADT exposure can also reduce T cell infiltration and major histocompatibility complex (MHC) class I expression, thereby diminishing immunotherapy’s efficacy^[52]^. Finally, mCRPC is characterized by a high degree of genetic heterogeneity, resulting in tumor clones with distinct genetic profiles and varying degrees of susceptibility to immunotherapy^[53-55]^.

### ICI monotherapy in PC

A phase 1 trial evaluated nivolumab, an anti-programmed cell death-1 (PD-1) inhibitor, in the treatment of patients with advanced cancers (NCT00730639) and found no significant objective response rates (ORRs) among those with PC^[56,57]^. Subsequently, the phase 1b KEYNOTE-028 trial evaluated pembrolizumab, an anti-PD-1 inhibitor, in the treatment of patients with PD-ligand 1 (PD-L1)-positive advanced PC, reporting an ORR of 17.4% and a disease control rate of 52.2%^[58]^. Next, the phase 2 KEYNOTE-199 trial examined mCRPC patients previously treated with docetaxel, noting low response rates to pembrolizumab (3%-5%), although some patients experienced durable responses (> 16 months)^[59,60]^. Interestingly, exploratory biomarker analysis revealed that patients with homologous recombination repair (HRR) alterations, such as BRCA1/2 or ATM mutations, exhibited higher response rates, with 11% showing improved outcomes. These findings suggest that genomic changes, including increased neoantigen burden and PD-L1 expression, may enhance tumor immunogenicity and improve ICI responses. Trials of single-agent ICIs using ipilimumab, an anti-cytotoxic T cell lymphocyte antigen-4 (anti-CTLA-4) inhibitor, have also demonstrated limited benefit in mCRPC^[5]^. Given the poor efficacy of single-agent ICI in mCRPC, there has been a shift toward combination approaches that may overcome drug resistance and improve antitumor immune responses through synergistic mechanisms.

The mismatch repair (MMR) pathway is responsible for DNA damage repair during replication^[61]^. Deficiencies in the MMR pathway (dMMR) in PC are highly correlated with increased neoantigen expression and a higher proportion of tumor-infiltrating lymphocytes^[62]^. These alterations, typically present in tumors with high TMB and microsatellite instability (MSI)^[63]^, often involve MSH2 and MSH6^[63]^. While MSI is found in only around 5% of mCRPC, dMMR or MSI-high (MSI-H) disease has shown a favorable response to ICIs, prompting pembrolizumab approval for patients with solid tumors (including PC) who have dMMR/MSI-H disease that is refractory to prior treatment, with no alternative treatment options^[64]^. Subsequent studies have further demonstrated the potential efficacy of pembrolizumab in this subset. For example, a single-center study found that 6/11 patients with dMMR/MSI-H mCRPC treated with PD-1/PD-L1-directed therapy had a > 50% PSA decline, of which 4 achieved a radiographic response^[65]^. Another single-center study observed > 50% PSA declines in 8/48 heavily pretreated patients with mCRPC^[66]^.

### ICI combination-based therapies in PC [Table 1]

**Table 1:** Summary of selected key ICI combination therapy trials in mCRPC

**NCT number**	**Phase**	**Estimated patients**	**Study description**	**Results**
NCT02985957^[67]^	2	90	A study of nivolumab + ipilimumab, ipilimumab alone, or cabazitaxel in men with mCRPC	Higher ORR (25%) in pre-chemotherapy cohort 1 *vs*. post-chemotherapy cohort 2 (10%). Grade 3-5 AEs were present in a significant proportion of study patients, with treatment-related deaths
NCT03016312^[69]^	3	772	Atezolizumab + enzalutamide *vs*. placebo + enzalutamide in mCRPC	Primary endpoint of OS was not met
NCT03834493^[70]^	3	1,244	Pembrolizumab + enzalutamide *vs*. placebo + enzalutamide in mCRPC	Primary endpoint of OS was not met
NCT03834519^[71]^	3	529	Pembrolizumab + olaparib for patients with previously treated and biomarker-unselected mCRPC	Primary endpoints of rPFS and OS were not met
NCT03834506^[74]^	3	1,030	Pembrolizumab + docetaxel *vs*. docetaxel in mCRPC	Primary endpoints of rPFS and OS were not met
NCT04100018^[76]^	3	984	Nivolumab + docetaxel *vs*. placebo + docetaxel in mCRPC	Primary endpoints of rPFS and OS were not met

ICIs: Immune checkpoint inhibitors; mCRPC: metastatic castrate-resistant prostate cancer; ORR: objective response rate; AEs: adverse events; OS: overall survival; rPFS: radiographic progression-free survival

The single-arm phase 2 CheckMate 650 trial investigated dual ICI treatment, nivolumab + ipilimumab, in patients with mCRPC. While an ORR of 25% was observed in the pre-chemotherapy cohort, grade 3/4 toxicity was observed in 42%-53% of treated patients, including four treatment-related deaths^[67]^.

The use of ICIs in combination with standard-of-care therapies has also been explored to induce a more inflamed TME and enhance antitumor immune responses. For instance, preclinical studies indicate the suppression of androgen levels may promote enhanced immune cell infiltration and delay CD8+ T cell (CTL) exhaustion^[68]^. Two phase 3 trials evaluated patients with mCRPC who received atezolizumab (anti-PD-L1)/enzalutamide (IMbassador250/NCT03016312) and pembrolizumab/enzalutamide (KEYNOTE-641/NCT03834493). However, neither combination demonstrated improved OS over enzalutamide alone^[69,70]^.

Next, pembrolizumab was tested in combination with olaparib in the phase 3 KEYLYNK-010 trial^[71]^. Olaparib, a PARPi approved for HRR gene-mutated mCRPC^[72]^, did not show improvement in radiographic progression-free survival (rPFS) or OS in that study. Consequently, the addition of chemotherapy to ICI was investigated in mCRPC, given the potential to boost the antitumor immune response^[73]^. Docetaxel is the only frontline chemotherapy approved for mCRPC based on improvement in OS^[19]^. In the phase 3 KEYNOTE-921 trial (NCT03834506), Docetaxel + pembrolizumab failed to outperform docetaxel alone, with no OS improvement^[74,75]^. Finally, in the phase 3 CheckMate-7DX trial (NCT04100018), treatment with nivolumab + docetaxel failed to improve rPFS or OS in patients with mCRPC^[76]^.

In summary, ICI combination regimens thus far have failed to demonstrate a definitive survival advantage in mCRPC. Overcoming immunotherapy resistance in mCRPC may require developing combinatorial approaches specific to individual immune phenotypes or genomic contexts, for which an improved understanding of the mechanisms of primary and acquired resistance will be crucial^[42]^. Several ongoing clinical trials are evaluating ICI combination therapies in mCRPC, including chemoimmunotherapy (NCT 04709276, NCT05563558) and ICI + targeted therapies (NCT04471974, NCT04848337, NCT05502315, NCT05445609, NCT04388852, NCT03568656, NCT05168618, NCT05176483, NCT04585750, NCT03866382, NCT05215574)^[77-89]^.

## CHEMOTHERAPY, RADIOPHARMACEUTICAL THERAPY, AND TARGETED THERAPY RESISTANCE MECHANISMS IN PC

### Chemotherapy resistance mechanisms

Docetaxel is an antimitotic taxane-based chemotherapeutic agent currently approved for first-line use in mCRPC^[19]^. Its mechanisms include inhibiting microtubule disassembly, reducing AR transcriptional activity, and suppressing B-cell lymphoma (BCL)-2 expression, thus promoting apoptosis^[90]^. Cabazitaxel is currently approved as a second-line treatment for mCRPC^[19]^. It is a semisynthetic taxane whose efficacy was demonstrated in the phase 3 TROPIC trial among patients previously treated with docetaxel^[91]^. Cabazitaxel inhibits microtubule disassembly and has a poor affinity for the ABCB1 protein, a cellular drug efflux pump, which decreases the likelihood of drug resistance. Additionally, it can cross the blood-brain barrier and block nuclear AR transport^[90]^. Reasons for acquired chemotherapy resistance include AR signaling reactivation, which promotes a pro-proliferative state. There is also upregulation of pro-survival pathways, including BCL-2, phosphoinositide 3-kinase (PI3K), and mammalian target of rapamycin (mTOR). Furthermore, the TME can hinder chemotherapy delivery due to the absence of lymphatic vessels and spherule formation by cancer cells^[90]^. Other common resistance mechanisms include increased ABCB1 leading to drug efflux, microtubule alterations mediated by β-tubulin mutations, and EMT, which allows PC cells to migrate or metastasize^[92,93]^.

### Ra-223 radionuclide therapy resistance mechanisms

Ra-223 is an α-particle-emitting radionuclide therapy (RNT) approved for patients with mCRPC with bony metastases^[19]^. Ra-223 preferentially incorporates into newly formed bone matrix within osteoblastic metastatic lesions and induces double-stranded DNA breaks, resulting in apoptosis of tumor cells, osteoblasts, and osteoclasts^[94]^. The phase 3 ALSYMPCA trial demonstrated an increase in OS in mCRPC patients with bony metastases treated with Ra-223^[95]^. Because of α-emitters’ unique mechanism of action, Ra-223 might be less susceptible to resistance mechanisms commonly encountered with other therapies^[94]^. While α-emitting RNTs such as Ra-223 remain an effective treatment for mCRPC, there are also potential challenges. First, supply and production are often limited as they are expensive to produce, heavily regulated, and vulnerable to supply-chain disruptions. Second, Ra-223’s efficacy is limited to patients with bony lesions. Third, there is a risk for increased toxicity, including bone marrow damage and secondary malignancies from radiation treatment (RT). Finally, calculating the appropriate RT dose can be difficult because these agents produce very few or no gamma rays, making them hard to track on conventional imaging^[96]^.

Novel α-emitting RNTs and combination approaches with standard-of-care therapies are currently being investigated in mCRPC to improve outcomes. Recently, the results of the phase 3 PEACE III trial (NCT02194842) were presented at the 2024 ESMO meeting. This study compared Ra-223 + enzalutamide with enzalutamide alone in patients with mCRPC and demonstrated a significant benefit for rPFS and an interim OS advantage, pending further data^[97]^.

### Prostate-specific membrane antigen RNT resistance mechanisms

Lutetium-177 vipivotide tetraxetan prostate-specific membrane antigen (PSMA) (177Lu-PSMA-617) is a β-particle-emitting RNT approved for use in patients with mCRPC who show disease progression after at least 1 ARSI and 1 or 2 taxane-based chemotherapy regimens^[19]^. It targets PC cells overexpressing the PSMA transmembrane protein and is used alongside a diagnostic PSMA positron emission tomography (PET) scan. Its clinical efficacy has been established in two pivotal clinical trials: (1) the phase 3 VISION trial, which showed PSMA-targeted RNT plus standard-of-care led to an increase in rPFS and OS in advanced PSMA-positive mCRPC^[98]^; and (2) the phase 2 Thera-P trial, which showed a higher PSA response rate for the PSMA arm compared to cabazitaxel^[99]^.

While 177Lu-PSMA-617 represents a promising treatment option for mCRPC, the duration of response is usually short-lived; up to 30% of patients have inherent resistance, and most patients eventually develop acquired resistance^[100]^. Potential resistance mechanisms include insufficient RT dose delivery due to low PSMA expression in tumor cells, as well as β-particles dose dependence on tumor size, so micrometastatic disease sites may receive inadequate RT. Additionally, the distribution of metastatic disease can affect RT response, likely due to site-specific TME variations: for example, nodal metastases demonstrated improved PSMA-based RT responses relative to osseous sites^[101]^. Hepatic metastases respond poorly to PSMA-based RT and show worse survival outcomes regardless of PSMA expression^[102]^. Finally, certain genetic alterations may impact RT response. For instance, TP53 mutations, present in up to 43% of PC tumors, have been associated with poor RT responses^[103,104]^.

Strategies to overcome PSMA β-emitting RT resistance may include optimizing patient selection, employing synergistic treatment combinations, and utilizing α-emitting RT, which can deliver a greater cytotoxic effect while minimizing normal tissue damage^[100,105]^. Preclinical data show that histone deacetylase (HDAC) inhibitors increase cell surface PSMA expression in mCRPC, potentially improving PSMA-RT effectiveness^[106]^. Similarly, the use of ICIs in combination with PSMA-RT could be beneficial since radiation-induced damage enhances the immunogenicity of tumor cells, possibly leading to better treatment outcomes^[107]^. Finally, combining PARPis with PSMA-RT may improve antitumor efficacy by co-inhibiting the DNA repair pathway, although preclinical results have been inconclusive so far^[108,109]^.

### PARPi resistance mechanisms

PARPis are a class of drugs designed to target tumors with defective DNA repair mechanisms. PARP-1 and PARP-2 are enzymes involved in single-stranded DNA break (SSB) repair. When PARPis are used in tumors with defective *HRR* genes, cells are incapable of repairing SSBs, resulting in apoptosis. PARPis can also trap PARP enzymes on DNA, destabilizing replication forks and promoting cell death in susceptible cells^[110]^. Olaparib is a PARPi approved in mCRPC with deleterious HRR mutations^[19]^. In the phase 3 PROfound study, OS improved for mCRPC patients with BRCA1/2 or ATM mutations^[72]^.

Potential PARPi resistance mechanisms include upregulated ABCB1 leading to drug efflux, restoration of HRR function through BRCA1/2 mutational reversions that restore the open reading frame, and stabilized stalled DNA replication forks, preventing further DNA damage by nucleases. In addition, PARP-1 mutations that block DNA trapping also contribute to PARPi resistance^[111]^.

Multiple studies employing CRISPR screens have identified DNA damage repair pathway gene mutations and tumor suppressor loss that contribute to PARPi sensitivity^[112,113]^. For instance, one genome-wide CRISPR screen in BRCA ½-proficient PC found cells with MMS2L gene deletion (an *HRR* gene) highly sensitive to PARPi. Loss of the DNA damage repair gene CHEK2 led to resistance against PARPi^[112]^. Another study demonstrated that loss of RB1 further decreased PARPi sensitivity in PC cell lines carrying a co-deletion of the DNA damage response gene RNASEH2B^[113]^. Collectively, these studies advanced our understanding of PARPi resistance mechanisms, and therefore, combination therapies aimed at overcoming PARPi resistance remain an active area of research. These include CDK inhibitors, which disrupt DNA replication fork stabilization and promote apoptosis, and epigenetic therapies like HDAC inhibitors, which may resensitize tumors to PARPi^[114]^.

### AR-INDEPENDENT RESISTANCE MECHANISMS IN PC

Several AR-independent signaling pathways contribute to drug resistance in mCRPC. Multiple single-cell studies have identified subsets of PC cells that drive disease progression following ADT and ARSIs^[115,116]^. For example, one study performed single-cell analysis of chromatin and RNA using PC cell lines previously treated with enzalutamide^[115]^. It revealed a subpopulation of enzalutamide-resistant PC cells characterized by high turnover and distinct transcriptional signatures associated with AR-targeted treatment response. Additional mechanisms of AR-independent resistance include NEPC, which is characterized by loss of AR expression and confers a poor prognosis^[117]^. Treatment options for NEPC patients who progress beyond platinum therapy are not well-established. 177Lu-PSMA-617 is currently being evaluated in a phase 2 trial (NCT05691465)^[118]^, and two trials (NCT05652686, NCT04471727) are evaluating therapies targeting delta-like protein 3, which is expressed on NEPC tumors^[119,120]^.

Another pathway involves the overexpression of growth factors, such as vascular endothelial (VEGF), fibroblast (FGF), and transforming growth-factor-beta (TGF-β). A phase 1 trial (NCT06457919) is evaluating tinengotinib in combination with standard-of-care therapies^[121]^. Additionally, a phase 2 study (NCT02452008) is examining the use of a TGF-β small-molecule inhibitor in combination with enzalutamide in mCRPC^[122]^. Increased activity of signaling cascades - Ras/mitogen-activated protein kinase (MAPK), PI3K/Ak strain transforming (AKT)/mTOR, Janus kinase (JAK)/signal transducer and activator of transcription (STAT), and Wnt-β catenin - which govern cell survival, division, and metabolism^[5]^, can also affect drug resistance. More specifically, PTEN aberrations (deletion or mutation) are present in 35% of mCRPC cases^[123]^. Loss of PTEN function leads to increased activity of downstream effectors (AKT and mTOR), driving tumor growth and correlating with a worse prognosis^[123]^. PI3K, AKT, and mTOR inhibitors have been evaluated as monotherapy in several early phase clinical trials, yet show limited efficacy^[5]^. This has prompted a shift to combination therapies. A phase III trial tested ipatasertib (AKT inhibitor) in combination with abiraterone and prednisolone in 1,101 mCRPC patients. Among 521 evaluable patients with PTEN loss, the combination yielded a significant improvement in rPFS compared to placebo + abiraterone, although there was no significant difference in the intention-to-treat cohort^[124]^. A phase I/II trial (NCT01485861) is evaluating ipatasertib or apitolisib (mTOR inhibitor) plus abiraterone in mCRPC^[125]^. A phase 1 trial (NCT06190899) is also studying gedatolisib, a pan-PI3K/mTOR inhibitor, in combination with darolutamide^[126]^. Finally, a phase 3 trial (NCT05348577) is evaluating capivasertib (an AKT inhibitor) + docetaxel *vs*. docetaxel alone in mCRPC^[127]^.

Additionally, the SRC signaling pathway (non-receptor tyrosine kinase) has been implicated in the development of CRPC and bone metastasis^[128]^. While preclinical studies demonstrated antitumor activity with SRC inhibitors, a phase III trial evaluating dasatinib, an SRC inhibitor, in combination with docetaxel failed to improve OS in mCRPC^[129]^. The Wnt/β-catenin pathway has also been implicated in PC pathogenesis^[130]^. Wnt binding to its receptor stabilizes β-catenin, increasing its concentration and driving transcription of genes promoting PC growth, treatment resistance, and neuroendocrine transformation^[5]^. Several Wnt inhibitors have been designed to block Wnt-receptor binding, prevent Wnt release intracellularly, or inhibit β-catenin’s interaction with the transcriptional coactivator CBP^[5]^. Currently, no active trials are evaluating Wnt inhibitors in mCRPC. A phase 1 trial (NCT05156905) had planned to test docetaxel + cirmtuzumab (a Wnt inhibitor), but was terminated prematurely^[131]^.

Next, gene alterations in CDK4/6, which regulate the cell cycle, can also lead to hyperproliferation. CDK4/6 controls the transition from G1 to S phase. In preclinical studies, CDK4/6 inhibitors can inhibit PC proliferation^[16]^. A phase 2 study (NCT04751929) assesses the use of abemaciclib +/- atezolizumab mCRPC^[132]^. Lastly, epigenetic modifications (acetylation and methylation) in histone proteins and DNA have been associated with PC progression^[5]^. Overexpression of EZH2, a histone methyltransferase leading to increased histone methylation, contributes to CRPC development and is under study as a potential drug target^[133]^. Several early phase trials are evaluating EZH2 inhibitors in combination with ARSI or ICI^[5]^. Additionally, histone acetyltransferases (HATs) such as p300 and CBP, which regulate AR transcription, can be inhibited by p300/CBP blockers, which are also under study. Conversely, HDACs decrease histone acetylation, and their elevated expression correlates with worse CRPC outcomes^[134]^. A phase 1 trial (NCT04703920) is evaluating talazoparib (a PARPi) + belinostat (an HDAC inhibitor)^[135]^. Finally, DNA methyltransferases (DNMTs) facilitate DNA methylation, leading to tumor suppressor gene silencing. A phase I/II study of azacitidine (a DNMT inhibitor) with docetaxel/prednisone in docetaxel-pretreated patients showed a PSA response in 10/19 and an ORR of 3/10 among evaluable participants^[136]^.

## FUTURE DIRECTIONS

Despite initial challenges, innovative therapeutic approaches to overcome drug resistance in mCRPC remain an active area of investigation. These include the use of bispecific antibodies (BsAbs), chimeric antigen receptor T cells (CAR T), AR degraders, RNTs, vaccines, antibody-drug conjugates (ADCs), targeted therapies, and novel ICI combination therapies [Table 2].

**Table 2:** Ongoing selected clinical trials focusing on innovative treatment approaches for mCRPC

**NCT number**	**Phase**	**Estimated patients**	**Invest. drug target (s)**	**Study description**	**Sponsor**
NCT02807805^[30]^	2	37	AR-V7	Abiraterone acetate, niclosamide, +prednisone in treating pts with CRPC	UC Davis
NCT05691465^[118]^	2	30	Somatostatin	Testing the safety and effectiveness of radiation-based Tx (Lu177 dotatate) for mNEPC	NCI
NCT05652686^[119]^	1/2	61	DLL3, CD47	Study of PT217 in pts with NEC expressing DLL3 (SKYBRIDGE Study)	Phanes therapeutics
NCT06190899^[126]^	1/2	54	PI3K, mTOR	Gedatolisib + darolutamide in mCRPC	Celcuity Inc.
NCT04751929^[133]^	2	75	CDK4/6,PD-L1	Abemaciclib +/-atezolizumab for mCRPC	DFCI
NCT05848011^[137]^	2	150	PD-1, CTLA-4	Study of lorigerlimab + docetaxel or docetaxel alone in pts with mCRPC	MacroGenics
NCT05005728^[138]^	2	85	PD-1, CTLA-4	XmAb®20717 (vudalimab) +/- chemo or targeted Txs in pts with mCRPC	Xencor, Inc.
NCT06193486^[139]^	1	30	PSCA	Autologous gamma delta T cells to target PSCA in mCRPC	Moffitt
NCT06228404^[140]^	1	18	PSMA	Clinical study of safety and efficacy of enhanced PSMA CAR T in refractory CRPC	Shanghai Changzheng Hospital
NCT06303713^[141]^	1	37	PSMA	LuCarbo - a study of 177Lu-PSMA-617 + carbo in mCRPC	DFCI
NCT04090528^[142]^	2	60	PD-1	pTVG-HP DNA vaccine +/- pTVG-AR DNA vaccine and pembro in pts with mCRPC	UW-Madison
NCT04662580^[143]^	1	262	PSMA	ARX517 in pts with mCRPC	Ambrx, Inc.

mCRPC: Metastatic castrate-resistant prostate cancer; Pts: patients; Invest: investigational; AR-V7: androgen receptor splice variant 7; Tx: treatment; Lu 177: Lutetium 177; NEPC: neuroendocrine prostate cancer; NEC: neuroendocrine cancer; DLL3: delta-like ligand 3; CD47: cluster of differentiation 47; PI3K: phosphoinositide 3-kinases; mTOR: mammalian target of rapamycin; CDK 4/6: cyclin-dependent kinase 4/6; PD-L1: programmed death-ligand 1; CTLA-4: cytotoxic T-lymphocyte-associated protein-4; chemo: chemotherapy; PSCA: prostate stem cell antigen; PSMA: prostate-specific membrane antigen; CAR T: chimeric antigen receptor T cell therapy; carbo: carboplatin; pTVG-HP: plasmid DNA encoding human prostatic acid phosphatase; pTVG-AR: plasmid DNA encoding androgen receptor ligand binding protein; pembro: pembrolizumab.

### BsAbs

**Figure 1:**
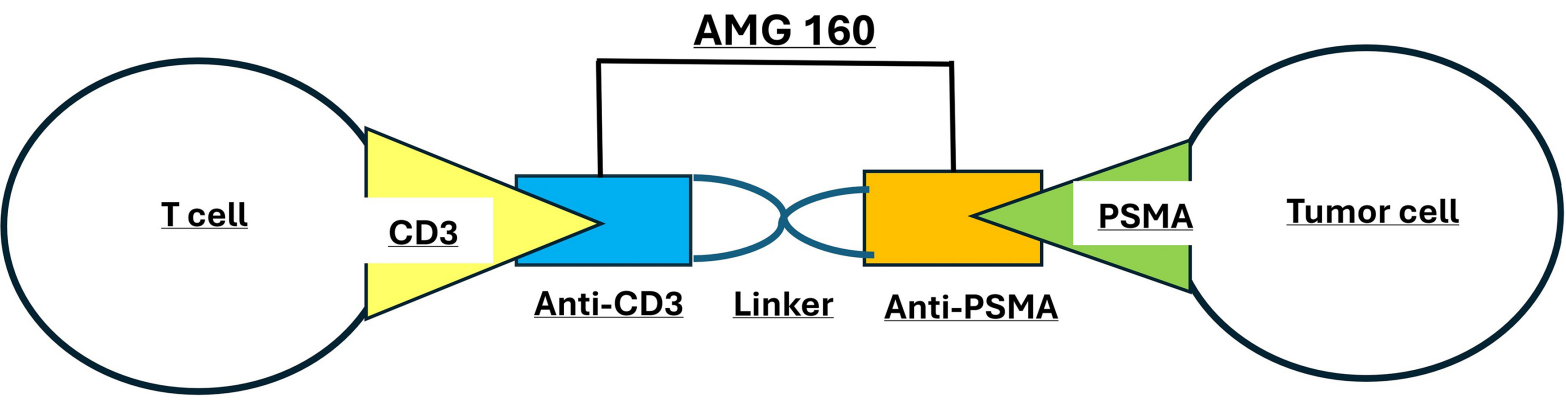
Schematic representation of AMG 160, a BiTE targeting PSMA on tumor cells and CD3 on T cells. By simultaneously binding to PSMA-expressing prostate cancer cells and CD3 on T cells, AMG 160 facilitates T cell-mediated cytotoxicity against tumor cells. BiTE: Bispecific T cell engager; PSMA: prostate-specific membrane antigen.

BsAbs are monoclonal antibodies (mAbs) capable of binding a tumor-associated antigen (TAA) as well as a CTL, through a costimulatory T cell receptor (TCR) such as clustered domain (CD) 3, CD28, or CD137, to bring the two cells into proximity^[144]^. Once binding occurs, T cell signaling pathways are activated, promoting an antitumor immune response. AMG 160 is an example of a bispecific T cell engager (BiTE) developed to direct cytotoxic T cells toward PC cells via PSMA targeting. As illustrated in Figure 1, AMG 160 contains two single-chain variable fragments (scFv): one specific for PSMA on the tumor cell surface and the other for CD3 on T cells, thereby bringing the two cell types into close proximity and enhancing T cell-mediated tumor cell killing. A variety of molecular constructs have been conceived to bolster BsAbs, including tandem scFv, dual-affinity retargeting (DART) molecules, and bi/tri-specific T cell engagers (BiTEs/TriTEs)^[145]^.

Challenges associated with BsAb therapy include short half-life, necessitating frequent dosing. Additionally, they carry risks for on-target and off-tumor toxicity and the development of severe adverse events (AEs) such as cytokine release syndrome (CRS). Moreover, there is a lack of sustained responses due to the development of drug resistance^[146-148]^.

A phase 1 trial (NCT03792841) evaluated AMG 160 in 43 patients with mCRPC^[149]^. Preliminary results (2020 ESMO) showed a manageable safety profile and several deep PSA and imaging responses in a heavily pretreated population. Next, a phase 1 trial evaluated AMG 340, another PSMA × CD3 BiTE, in 41 patients with mCRPC. Of 27 evaluable patients, 14 experienced stable disease per RECIST, and the ORR was 0^[150]^. Of note, both therapies have since been discontinued. The phase Ia ENGAGER-PSMA-01 trial (NCT05519449) is currently evaluating JANX007, a novel PSMA x CD3 BiTE, in mCRPC. Initial results were recently disclosed in a press release, in which JANX007 demonstrated significant antitumor activity in 16 heavily pretreated patients (median prior lines of therapy = 4), with a PSA50 response rate of 100%. Among 8 RECIST-evaluable patients, 50% achieved a partial response^[151]^.

Another phase 1 trial investigated lorigerlimab, a PD-1 × CTLA-4 DART BsAb, in mCRPC. Treatment-related AEs (TRAEs) occurred in 85.8% (109/127) of participants and led to discontinuation in 22.8%^[137]^. The study reported an ORR of 25.7% (9/35) and a PSA50 of 28.6% (12/42). Several early-phase BsAbs trials are ongoing, including longerlimab + chemotherapy (NCT05848011), a BsAb targeting human kallikrein 2 (NCT04898634), a human epidermal growth factor receptor (HER)2 × HER3 BsAb (NCT05588609), and a PD1 × CTLA-4 BsAb +/- chemotherapy or targeted therapy (NCT05005728)^[138,152-154]^. Additionally, two ICI + BsAb trials are underway (NCT05733351, NCT05369000)^[155,156]^.

### CAR T cell therapy

**Figure 2:**
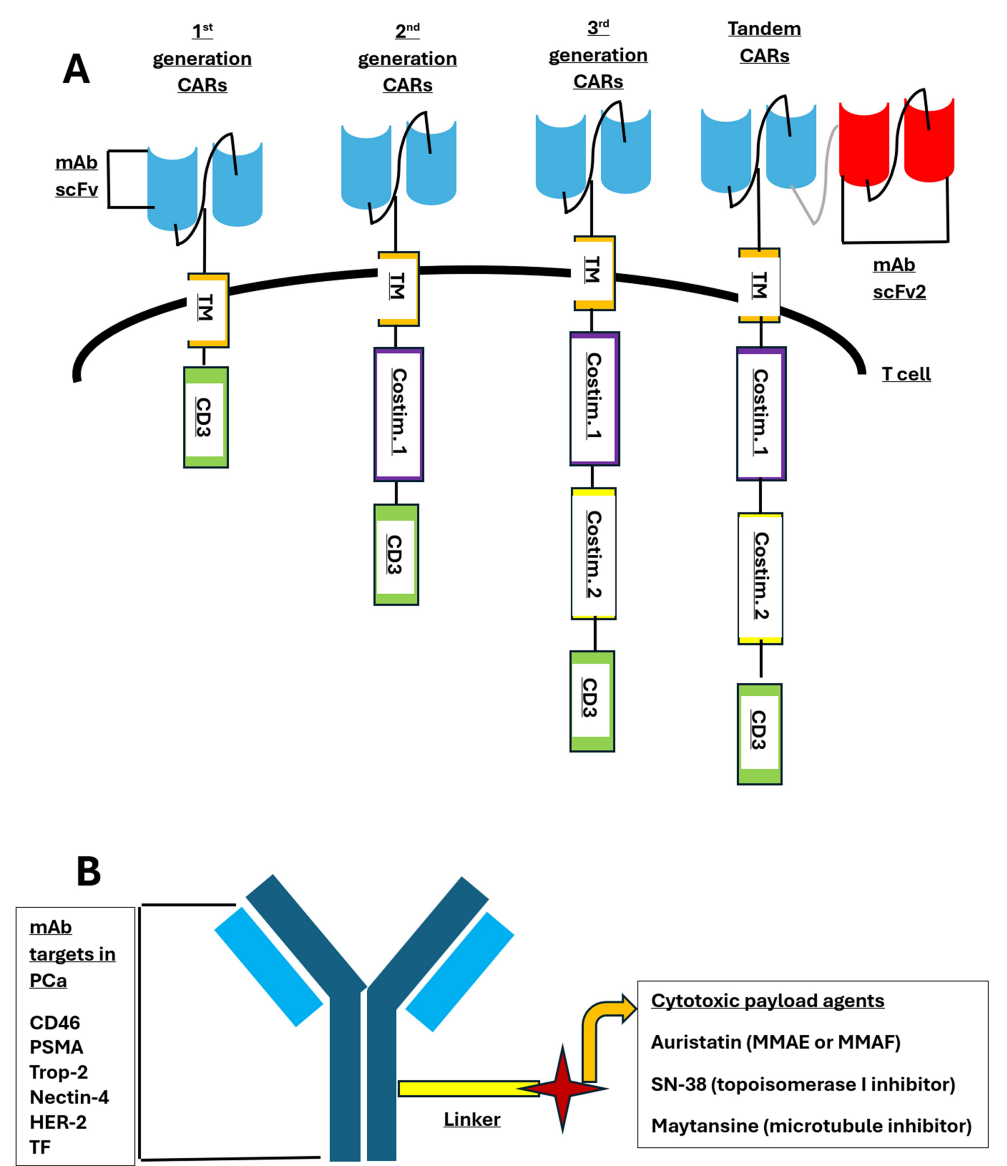
Structures of CAR T cells and ADCs in prostate cancer therapy. (A) Evolution of CAR T cell designs across generations. First-generation CARs only contain the CD3 signaling domain. Second-generation CARs add one costimulatory domain (Costim 1). Third-generation CARs incorporate two costimulatory domains (Costim 1 and 2). Tandem CARs can target two different antigens simultaneously through dual scFv regions. All generations include an extracellular antigen-binding domain (mAb scFv) and TM; (B) Basic structure of ADCs showing monoclonal antibody targets in PCa and associated cytotoxic payload agents connected via a linker. CAR: Chimeric antigen receptor; ADCs: antibody-drug conjugates; scFv: single-chain variable fragments; mAbs: monoclonal antibodies; TM: transmembrane domain; Pca: prostate cancer; PSMA: prostate-specific membrane antigen.

CAR T cell therapy is a type of adoptive cell therapy using the genetic engineering of autologous T lymphocytes to express TAA-specific synthetic receptors^[157]^. As shown in Figure 2A, first-generation constructs consist of an extracellular target-binding domain derived from a tumor-specific mAb scFv and an intracellular CD3 T cell signaling domain that activates T cell effector functions^[157,158]^. Key roles for CAR T cells are TAA recognition in an MHC-independent manner, T cell activation and proliferation, and cytotoxic activity against malignant cells^[159]^. Subsequent CAR generations include several intracellular domains, which improve antitumor activity in preclinical studies^[160]^. While CAR T cell therapy succeeds in hematologic malignancies, its efficacy in solid tumors has been limited thus far^[161]^.

Primary therapeutic CAR T cell targets in mCRPC include prostate stem cell antigen (PSCA) and PSMA. A phase 1 study (NCT03873805) evaluated the safety and efficacy of PSCA-directed CAR T cells. Grade 1/2 CRS occurred in 5/14 treated patients and PSA30 responses were noted in 4/14 patients^[162]^. Dynamic changes indicating the activation of peripheral blood endogenous and CAR T cell subsets, TCR repertoire diversity, and TME changes were observed in some patients, though CAR T cells beyond 28 days post-infusion were limited. Additionally, a phase 1 study (NCT03089203) evaluated PSMA-targeting TGF-β-insensitive “armored” CAR T cells, which modulate the immunosuppressive TME in PC by acting as a TGF-β sink, thereby improving CAR T efficacy^[163]^. This study reported 5/13 patients developing grade ≥ 2 CRS and 4 achieving a PSA30 response. Notably, one patient demonstrated significant clonal CAR T expansion and a 98% PSA reduction but unfortunately died of grade 4 CRS complicated by concurrent sepsis^[164]^.

A preclinical study using mouse models demonstrated antitumor activity in PC cells treated with CAR T targeting the six transmembrane epithelial antigens of the prostate-1 (STEAP-1)^[165]^. Currently, a phase I/II trial (NCT06236139) is evaluating STEAP-1-directed CAR T therapy with enzalutamide in mCRPC. Challenges with CAR T include poor trafficking to tumor sites, an immunosuppressive TME, and limited cell persistence^[166]^. Additionally, there is an increased risk of severe drug-related AEs and off-tumor toxicity, including CRS, tumor lysis syndrome, macrophage activation syndrome, and neurologic and cardiovascular toxicities^[161]^. CRS typically occurs within 1-14 days following treatment, and is frequently reported (57%-93% of patients)^[167]^. Common manifestations of CRS include fever and hypotension, which can be fatal if untreated. A standardized grading scale guides management, with severe cases requiring IV steroids^[168,169]^. Finally, there is a risk of drug resistance through antigen escape^[158]^.

Strategies to enhance CAR T therapy efficacy in mCRPC may include combinatorial approaches to overcome immunosuppressive signals and developing tandem CARs that target several TAAs simultaneously. For example, a preclinical study combined IL-12 with STEAP-1-directed CAR T, yielding improved antitumor immune response^[165]^. Additionally, chemokine receptors can be added to improve CAR T delivery to tumor sites, as shown in several preclinical studies^[170]^. Finally, to reduce the risk of off-tumor toxicity, an “off switch” can be incorporated. For instance, synthetic Notch (SynNotch) circuits require recognition of a “priming antigen” on the target cell before CAR T-mediated cytotoxicity^[171]^. Several ongoing trials are investigating approaches to overcome CAR T cell resistance, including PSCA- (NCT06193486, NCT05805371) and PSMA-targeted (NCT06228404) therapies^[139,140,172]^.

### ADCs

ADCs have demonstrated benefit in several malignancies, including urothelial carcinoma, and are now under investigation for mCRPC. As illustrated in Figure 2B, ADCs consist of a mAb that binds a cell surface antigen plus a cytotoxic payload that induces tumor cell death upon internalization. These components are connected by a stable linker cleavable at the target site^[173]^.

An advantage of ADCs is their direct delivery into cancer cells, maximizing efficacy and minimizing systemic toxicity. Additionally, the “bystander effect” enables antitumor activity regardless of antigen expression once the drug is released from targeted cells into the TME^[174]^. ADC targets in mCRPC under current evaluation include PSMA (NCT04662580), trophoblast cell-surface antigen 2 (NCT03725761, NCT05489211, NCT04644068), nectin-4 (NCT04754191), HER2 (NCT06227156), and CD46 (NCT05011188)^[143,175-180]^. ICI + ADC (NCT04925284) is also being evaluated^[181]^.

### AR protein degraders

**Figure 3:**
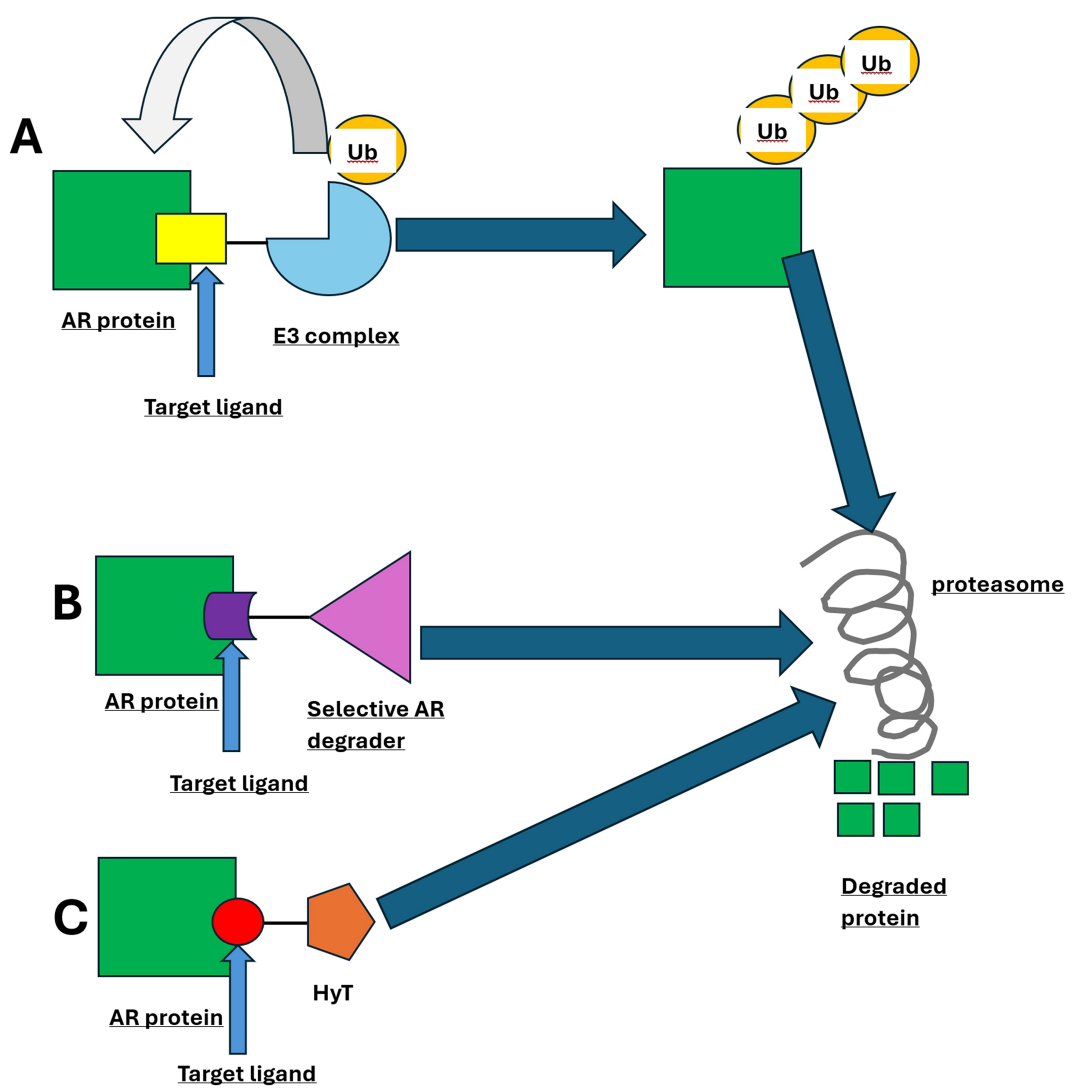
Mechanisms of AR protein degradation. (A) PROTACs recruit the AR protein and E3 Ub ligase complex into close proximity, facilitating ubiquitination and subsequent proteasomal degradation; (B) SARDs bind directly to AR and promote its degradation through the proteasome, reducing AR-mediated signaling; (C) Hydrophobic tagging involves attaching a lipophilic moiety to the AR protein, causing it to fold improperly and be recognized by the cell’s quality control machinery, ultimately targeting it for Ub-proteasome-mediated degradation. AR: Androgen receptor; PROTACs: proteolysis-targeting chimeras; Ub: ubiquitin; SARDs: selective androgen receptor degraders.

AR protein degradation represents a promising novel approach to overcoming mCRPC drug resistance. Such degraders can overcome several resistance mechanisms associated with AR antagonists, including AR point mutations, overexpression, and splice variants. Physiologically, the AR protein undergoes degradation via the Ub-proteasome system or via phosphatase and tensin homolog (PTEN)-caspase-3. Several AR protein degraders have been developed, including proteolysis-targeting chimeras (PROTACs), selective AR degraders (SARDs), and hydrophobic tagging, each employing a distinct mechanism [Figure 3].

PROTACs facilitate endogenous AR protein degradation, mediated via the E3 complex (part of the Ub enzymatic cascade). This results in the formation of a ternary complex, bringing the target protein and the Ub-proteasome system into close proximity^[182]^. Some key advantages of PROTACs include their high selectivity, the ability of a single molecule to degrade multiple proteins (resulting in higher drug potency), and their versatility in targeting proteins previously considered “undruggable” by small-molecule inhibitors^[183,184]^.

Preliminary findings from a phase 2 clinical trial (NCT03888612) evaluating ARV-110 (a PROTAC) in mCRPC patients were presented at the 2022 American Society of Clinical Oncology Genitourinary Cancers Symposium (ASCO GU). Nearly 50% of patients with T878X and H875Y mutations experienced a significant PSA reduction^[185]^. Moreover, initial results from a phase I/II trial evaluating ARV-766 (NCT05067140), another PROTAC, in 123 mCRPC patients previously treated with novel hormonal agents, were presented at the 2024 ASCO annual meeting, showing a PSA50 response rate of 43% among 47 evaluable patients with AR LBD mutations^[186]^. Additional PROTACs currently under investigation include CC-94676 (NCT04428788)^[187]^ and HP-518 (NCT05252364)^[188]^.

Next, SARDs are small molecules that bind specific AR domains, showing efficacy in reducing the activity of both wild-type and AR-Vs. Notably, several SARDs (UT-69 and UT-155) have demonstrated antitumor efficacy and, in one study, were more potent than enzalutamide in PC cell lines and mouse models^[189]^. Additionally, Z15, a novel dual-function SARD (AR antagonist and AR degrader), has shown potential efficacy in both *in vitro* and *in vivo* models^[190]^. UT-34 is another dual-function SARD that appears promising for enzalutamide-resistant CRPC. In preclinical work, it inhibited wild-type and LBD-mutant AR while also suppressing enzalutamide-resistant PC cell line growth^[191]^. A phase 1/2 trial (NCT05917470) had planned to evaluate UT-34 in mCRPC but was terminated for futility^[192]^.

Finally, hydrophobic tagging promotes AR protein degradation through Ub proteolysis. It involves attaching a bulky lipophilic component to the target protein, forming an unstable protein structure that mimics a denatured protein. Once recognized by cellular quality control, this hydrophobic tag triggers the unfolded protein response and targets the protein for degradation^[193]^. However, given the challenges of drug design, hydrophobic tagging degraders have yet to reach clinical testing^[194]^.

### RNTs

Actinium-225 (^225^Ac) PSMA is a novel RNT for patients with mCRPC. It is an α-targeted therapy characterized by high linear-energy transfer and a shorter path length, enabling more precise RT delivery to the tumor^[195]^. A recent multicenter retrospective study investigated ^225^Ac-PSMA-617 in mCRPC patients who had received one or more previous lines of treatment^[196]^. Among them, 70% experienced a PSA response, and 57% demonstrated a PSA50 response. Several clinical trials are evaluating PSMA-targeted RNT combination therapies in mCRPC^[141,197-204]^.

### Vaccines

Cancer vaccines represent a promising therapeutic approach in PC, designed to elicit robust tumor-specific CD4+ and CTL responses via recognition of tumor cells (non-antigen-specific) or TAAs (antigen-specific)^[205,206]^. Sipuleucel-T (Provenge) is an autologous, antigen-specific dendritic cell (DC) vaccine composed of a recombinant fusion protein, prostatic acid phosphatase (PAP), linked to granulocyte-macrophage colony-stimulating factor (GM-CSF), expressed on the surface of antigen-presenting cells (APCs)^[206]^. It was approved for asymptomatic or minimally symptomatic mCRPC following results from the phase 3 IMPACT trial^[43]^. Beyond DC vaccines, other types of vaccines studied in PC include GVAX (a GM-CSF-secreting cellular vaccine)^[207,208]^; a human leukocyte antigen (HLA)-A24 matched peptide-based vaccine^[209]^; PROSTVAC-VF/TRICOM, a viral vector-based vaccine^[210]^; and DNA-based vaccines targeting PSA, PAP, or AR antigens^[211-213]^. Thus far, sipuleucel-T is the only immunotherapy demonstrating improved OS in mCRPC^[43]^.

Messenger RNA (mRNA) vaccines use synthetic mRNA to produce a specific protein antigen capable of eliciting both innate and adaptive antitumor response^[214]^. A phase I/IIa trial (EudraCT number 2008-003967-37) evaluated CV9103, an mRNA vaccine (PSA, PSCA, PSMA, and STEAP1 antigens) in 44 patients with advanced CRPC^[215]^. Among 33 evaluable patients, 26 elicited an immune response, and 1 experienced a PSA response. Next, a phase I/IIb trial (EudraCT number: 2011-006314-14) tested CV9104, an mRNA vaccine (PSA, PSMA, PSCA, STEAP1, PAP, and MUC1 antigens) in 197 patients with mCRPC^[216]^. However, this study failed to demonstrate any improvement in rPFS or OS.

Vaccines in combination with other therapies will likely be needed to overcome drug resistance. For instance, combining plasmid DNA vaccines with anti-PD-1 ICIs has shown improved response rates^[217,218]^. Two trials are currently evaluating DNA-based vaccines + nivolumab (NCT03600350) and pembrolizumab (NCT04090528) in patients with nonmetastatic PC and mCRPC, respectively^[142,219]^. Several ICI + vaccine trials are also underway (NCT03532217, NCT02649855, NCT04989946)^[142,220,221]^.

## CONCLUSION

In summary, the mechanisms driving PC growth and contributing to drug resistance are complex, involving multiple signaling pathways. While immunotherapy has recently emerged as an innovative strategy in PC, its efficacy remains limited by low TMB and an immunosuppressive TME. The emergence of numerous novel treatments, including BsAbs, CAR T, AR degraders, and ADCs, provides further optimism for improved outcomes. As our understanding of underlying mechanisms grows, future research should focus on identifying reliable biomarkers to predict which patients benefit most and who may develop resistance. Further work is also needed to determine which regimens are effective in mCRPC through an individualized approach based on clinicopathological factors and tumor molecular profile. Moreover, adopting reliable predictive biomarkers may enable better selection of first- and subsequent-line therapies (including combinatorial regimens), and inform optimal sequencing of treatments in patients with mCRPC, an area where limited data currently exist. Rationally designed combination therapies, guided by biomarkers and mechanistic insights, can identify synergistic agents targeting multiple resistance pathways simultaneously while minimizing toxicities and improving long-term outcomes. Finally, careful evaluation of trial inclusion criteria for heavily pretreated populations will be crucial to maximize treatment benefits.

## DECLARATIONS

### Copyright

© The Author(s) 2025.
